# Breast Carcinoma Metastasis to the Medial Rectus Muscle: Case Report

**DOI:** 10.4274/tjo.galenos.2018.39018

**Published:** 2019-06-27

**Authors:** Özge Yabaş Kızıloğlu, Fatma Paksoy Türköz, Özgün Melike Totuk Gedar, Mert Mestanoğlu, Özlem Yapıcıer

**Affiliations:** 1 Bahçeşehir University Faculty of Medicine, Department of Ophthalmology, İstanbul, Turkey; 2 Bahçeşehir University Faculty of Medicine, Department of Internal Medicine, İstanbul, Turkey; 3 Bahçeşehir University Faculty of Medicine, İstanbul, Turkey; 4 Bahçeşehir University Faculty of Medicine, Department of Pathology, İstanbul, Turkey

**Keywords:** Breast carcinoma, extraocular muscle, medial rectus muscle, orbital metastasis

## Abstract

A 63-year-old woman with metastatic breast carcinoma presented to the ophthalmology clinic with diplopia and right abduction deficit. Magnetic resonance imaging showed isolated enlargement of the right medial rectus muscle. Biopsy of the enlarged muscle revealed metastasis of breast carcinoma. Ocular motility deficit in a patient with breast carcinoma should raise suspicion of metastasis to the orbit involving the extraocular muscles. Orbital imaging and biopsy are necessary for diagnosis and appropriate treatment.

## Introduction

The orbit is an unusual site for metastasis, being involved in 2 to 3% of cancer patients.^1^ The most prevalent primary tumor metastasizing to the orbit is breast carcinoma, which accounts for 28.5-58.8% of all orbital metastases.^2,3,4^

Orbital metastasis can present as the initial manifestation of breast carcinoma; however, in most cases, there is a previous history of breast cancer that has been treated, or an orbital mass occurs in a patient with active malignancy affecting multiple organs.^1^ Orbital breast carcinoma metastases may localize within orbital fat, bone, or extraocular muscles. Scirrhous infiltration of the orbit can also occur; resulting in enophthalmos.^5^ Definite diagnosis of orbital metastasis can be made by biopsy of the affected tissue. 

Orbital metastasis of breast carcinoma involving single or multiple extraocular muscles is infrequently diagnosed and has been reported in a small number of studies.^6,7,8,9,10,11^The purpose of this report is to describe a patient with metastatic involvement of the medial rectus muscle by breast carcinoma and to discuss related literature on orbital metastasis of breast carcinoma.

## Case Report

A 63-year-old woman with metastatic breast carcinoma presented to the ophthalmology clinic with diplopia in right gaze and head turn to the right. Medical history revealed that she was diagnosed with estrogen receptor (ER)-positive and progesterone receptor (PR)-positive invasive ductal carcinoma 1 year earlier with mediastinal lymph node and bone metastasis at the time of diagnosis. She was treated with zoledronic acid 4 mg monthly and paclitaxel 80 mg/m^2^ weekly for 12 weeks, followed by endocrine therapy with letrozole.

**Figure 1:**
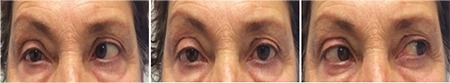
Images of the patient at presentation. Abduction of the right eye is limited with retraction of the globe and narrowing of the palpebral fissure

**Figure 2:**
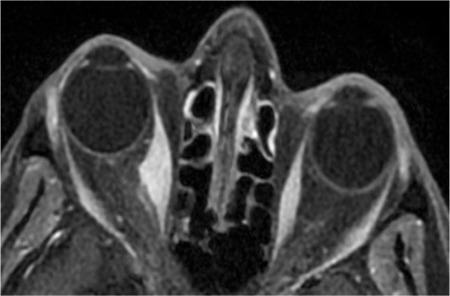
Magnetic resonance imaging of the orbit. Axial postcontrast T1- weighted image showing thickening of the right medial rectus muscle with sparing of the tendon

On ophthalmological examination, best corrected visual acuity was 20/25 in both eyes. Slit-lamp examination of the anterior segment and fundus was unremarkable other than bilateral posterior chamber intraocular lenses. On motility exam, abduction was totally limited in the right eye with globe retraction and narrowing of the palpebral fissure on attempted abduction (Figure 1). Abnormal head position towards the right side was noted. Magnetic resonance imaging (MRI) revealed isolated enlargement of the right medial rectus muscle (Figure 2). Clinical evaluation and laboratory studies were carried out for differential diagnosis. There were no clinical findings suggestive of thyroid eye disease and thyroid function tests were normal. Rheumatologic assessment for inflammatory and vasculitic diseases was not contributory. Biopsy of the right medial rectus muscle was performed to establish a definite diagnosis and initiate appropriate treatment. 

**Figure 3:**
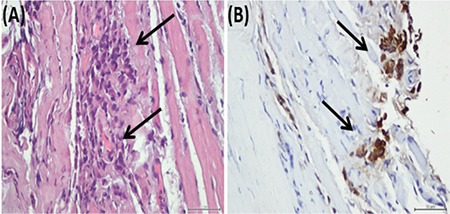
(A) Hematoxylin-eosin stain. The biopsy specimen of the right medial rectus muscle showing tumor cells (arrows) infiltrating muscle fibers; 400x. (B) Cytokeratin 7 immunohistochemistry of the specimen showing tumor cells (arrows); 400x

Hematoxylin and eosin staining of the biopsy specimen revealed large, round to polygonal epithelioid tumor cells arranged in loosely cohesive clusters and sheets infiltrating fibrocollagenous tissue and muscle fibers (Figure 3A). Immunohistochemical analyses using streptavidin-biotin peroxidase complex method revealed panCytokeratin and cytokeratin 7 positivity (Figure 3B). ER, PR and human epidermal growth factor receptor 2 (HER2/neu) were negative (triple-negative). Based on the patient’s clinical history and the morphological and immunohistochemical features of the tumor, she was diagnosed with breast carcinoma metastasis to the right medial rectus muscle. Pathological examination demonstrating a triple-negative breast carcinoma indicated discordance with the primary tumor, which was ER- and PR-positive at the time of diagnosis.

The patient was referred to the radiation oncology department for external beam radiation therapy. The orbital mass was irradiated with 45 Gy in 15 fractions. Following radiotherapy, chemotherapy with docetaxel 100 mg/m^2^ once every 21 days was initiated. After 15 months of follow-up, abduction of the right eye has partially recovered; the patient is stable and continuing to receive palliative chemotherapy.

## Discussion

Among all orbital tumors, metastatic cancer has a prevalence of 1-13%.^1^ The majority of ocular and orbital metastases are caused by breast cancer.^12^ The reported incidence of breast cancer metastasis to the ocular structures in clinical series varies between 8 and 10%. However, its incidence may be underestimated because of the concurrent involvement of major organs like lungs, liver, or bone, which may have more serious consequences dominating the patient’s clinical situation. 

Extraocular muscles are rarely infiltrated by metastatic tumors from distant sites. The rarity of extraocular muscle involvement by metastases has been attributed to the constant movement of these muscles, which prevents lodging of neoplastic cells, and to their unfavorable chemical environment for neoplastic growth.^13^ On the other hand, orbital metastases of breast carcinoma have a tendency to spread to the extraocular muscles and surrounding orbital fat.^5^ With the advancement of treatment options and prolonged survival of breast carcinoma patients, the possibility of extraocular muscle metastases of breast carcinoma may increase.^14^

Diplopia and ocular motility disorder in a patient with neoplastic disease should initially raise suspicion of tumor involvement of extraocular muscles; however, broad differential diagnosis is required to determine the cause and to institute appropriate treatment. Imaging with computed tomography or MRI is helpful in demonstrating extraocular muscle enlargement and determining extent of orbital involvement. Laboratory studies should be carried out to exclude other conditions that may cause extraocular muscle enlargement like granulomatous, vasculitic, endocrine, and immunologic diseases. Biopsy of the involved tissue is necessary for definite diagnosis.

In breast carcinoma cases, discordance of ER, PR and HER2/neu status between the primary tumor and subsequent metastases is well recognized.^15^ Several studies have shown substantial discordance rates between primary breast carcinoma and metastatic disease, reporting hormone receptor discordance rates between 30% and 40%.^16,17,18^ The primary tumor in our patient was ER-/PR-positive. However, biopsy and immunohistochemical staining of the metastatic lesion in the medial rectus muscle demonstrated triple-negative breast carcinoma, indicating discordance with the primary tumor. The result of the metastatic biopsy led to the modification of our treatment from endocrine therapy to chemotherapy. 

The time interval between diagnosis of primary breast carcinoma and detection of orbital metastasis is usually long; the mean interval has been reported to range from 4.5 to 6.5 years.^14^ In the current case, the orbital metastasis was diagnosed 1 year after the primary tumor, a relatively short interval in comparison to previous reports.

Treatment of orbital metastatic lesions may help to control the growth of the tumor, to preserve visual function, and to improve patient comfort. External-beam radiotherapy to the orbital metastatic lesion is the mainstay treatment.^5^ Chemotherapy and hormone therapy are other options, depending on the status of the systemic disease. The prognosis of breast carcinoma with orbital metastases is poor; survival ranges from 1 to 116 months with a mean of 31 months.^4^

In conclusion, ocular motility deficit in a patient with breast carcinoma should raise suspicion of a possible orbital metastatic lesion involving the extraocular muscles. Biopsy is required for definite diagnosis. The metastatic lesion may show discordance from the primary tumor, which may alter treatment decisions and follow-up of the disease.
